# Persistent Hypocalcemia Following Scopinaro Biliopancreatic Diversion: A Case Report

**DOI:** 10.7759/cureus.96549

**Published:** 2025-11-11

**Authors:** Yahya H Alqahtani, Mayyas Alnajmi, Esam Batayyah, Ahmed Qadah, Hanin M Attar, Raghad Hibah

**Affiliations:** 1 General Surgery, Al-Noor Specialist Hospital, Makkah, SAU; 2 General Surgery, King Abdullah Medical City, Makkah, SAU

**Keywords:** bariatric surgery complications, biliopancreatic diversion, persistent hypocalcemia, refractory hypocalcemia, scopinaro bypass

## Abstract

Bariatric surgery is a treatment for morbid obesity and related comorbidities; however, it carries risks of long-term nutritional deficiencies. Among the malabsorptive procedures, the Scopinaro biliopancreatic diversion (BPD) achieves durable weight loss but is associated with significant metabolic and nutritional complications, particularly those involving calcium, iron, and fat-soluble vitamins. This case report highlights a patient who developed severe, chronic hypocalcemia after undergoing Scopinaro BPD and ultimately required surgical conversion to a Roux-en-Y gastric bypass (RYGB) for resolution of his symptoms.

We present the case of a 50-year-old male with a history of morbid obesity who initially underwent gastric banding, followed years later by a Scopinaro BPD. Since the latter surgery, he experienced recurrent severe hypocalcemia, vitamin D deficiency, and iron-deficiency anemia, requiring repeated hospitalizations for intravenous calcium supplementation, iron therapy, and blood transfusions, despite strict adherence to oral supplementation. Five years after the procedure, he first presented with symptomatic anemia (hemoglobin, or Hgb 7 g/dL), managed with transfusion and iron therapy. Over the following decade, he experienced multiple similar admissions, including one with profound hypocalcemia (1.1 mmol/L), accompanied by dizziness, limb stiffness, and paresthesia. Workup for systemic causes, including hypoparathyroidism, was negative, and chronic malabsorption was determined to be the underlying etiology. After optimization of electrolytes and Hgb levels, the patient underwent laparoscopic takedown of the Scopinaro anastomosis with conversion to an RYGB. The surgery was completed without complication. Postoperatively, his calcium and Hgb improved steadily, reaching 1.9 mmol/L and 12 g/dL, respectively, at three months’ follow-up. He remained asymptomatic and demonstrated weight regain, with significant improvement in quality of life.

In our case, persistent hypocalcemia and anemia refractory to conservative treatment were successfully managed by conversion to RYGB, which restored absorptive capacity while maintaining a balance of weight control and improved nutritional status. This highlights the critical role of surgical revision in managing long-term metabolic complications of highly malabsorptive procedures.

## Introduction

Bariatric surgery is frequently associated with the development of postoperative vitamin D deficiency. This is largely due to anatomical and physiological alterations in the gastrointestinal (GI) tract that impair the absorption of several nutrients, particularly vitamin D and calcium. Vitamin D plays a crucial role in maintaining calcium homeostasis and bone metabolism, both essential for preserving bone health [1]. The complications resulting from vitamin D deficiency can be mitigated with routine supplementation post-surgery.

Preoperative vitamin D deficiency affects between 31.6% and 92% of morbidly obese individuals, particularly those undergoing sleeve gastrectomy. Therefore, regular postoperative monitoring of vitamin D, parathyroid hormone (PTH), and serum calcium levels is essential to ensure appropriate dosing and prevent deficiencies [2,3].

The effects of metabolic surgery on calcium homeostasis and skeletal health remain poorly understood. Intact intestinal mucosa may suggest preserved nutrient absorption, particularly in procedures like sleeve gastrectomy. However, research has shown that bone loss following these procedures may still occur, although it is typically less than that seen after Roux-en-Y gastric bypass (RYGB) [4-6]. 

The literature also highlights that the prevalence of hypocalcemia post-bariatric surgery varies significantly based on the surgical technique. It has been reported as low as 1% following RYGB, while reaching up to 25% after biliopancreatic diversion with duodenal switch (BPD-DS). The BPD was first described by Scopinaro in 1979. This procedure combined a horizontal gastric resection with the closure of a duodenal stump, a gastroileal anastomosis, and an ileoileal anastomosis to create a 50-cm common channel and a 250-cm alimentary channel. In this report, we present a case of severe hypocalcemia following a Scopinaro BPD, which resolved following surgical conversion to an RYGB.

## Case presentation

We present the case of a 50-year-old male who underwent gastric banding surgery 20 years ago. The band was removed three to four years later, and he subsequently underwent a Scopinaro BPD. Since this procedure, the patient has suffered from severe hypocalcemia, vitamin D deficiency, and iron-deficiency anemia, which have required multiple hospital admissions. These admissions involved blood transfusions and intravenous calcium, despite ongoing supplementation with oral calcium. 

The patient’s first admission was five years after the Scopinaro BPD, presenting with a hemoglobin (Hgb) level of 7 g/dL. He was transfused with two units of packed red blood cells (PRBCs) and was discharged on an iron supplement. The patient remained stable for the following two years, until he was diagnosed with mild hypocalcemia that was responsive to oral supplementation. Three years later, he experienced another severe drop in Hgb, which was treated with a transfusion of two units of PRBCs. 

Upon presentation to the Emergency Department, the patient complained of dizziness, stiffness, and numbness in his upper and lower limbs; he denied any chest pain. His vitals were all within normal range. Laboratory investigations revealed a calcium level of 1.1 mmol and Hgb of 10.5 g/L. He was admitted under the care of internal medicine, where he received intravenous calcium, ferric carboxymaltose (Ferinject), and blood transfusions. An investigation for other systemic causes, such as hypoparathyroidism, was negative, and he was diagnosed with chronic malabsorption.

The decision was made to proceed with surgical intervention, as the patient had been symptomatic for over a decade. After the patient’s electrolyte imbalances and Hgb level were adequately corrected, he was taken to the operating room for a laparoscopic takedown of the previous anastomosis with conversion to an RYGB. The procedure was performed without complications. 

Postoperatively, the patient's calcium and Hgb levels were monitored daily, with notable improvements reaching levels of calcium 1.9 mmol and Hgb 12.0 g/L before his discharge. The patient presented to the clinic three months later for a follow-up, where he was asymptomatic and had gained some weight postoperatively.

## Discussion

In 1979, Dr. Nicola Scopinaro performed the first BPD procedure at the University of Genoa in Italy, a modification of the gastric diversion procedure originally introduced by Dr. Edward Mason in 1966. Scopinaro’s procedure achieved significantly greater weight loss and was a revolutionary development in the field of metabolic surgery [7].

Although Scopinaro BPD is more effective and offers greater long-term weight loss durability than RYGB, it has significant disadvantages. A higher incidence of nutritional deficiencies - particularly of fat-soluble vitamins, calcium, and iron - is observed in patients who have undergone the Scopinaro BPD [8].

As our case demonstrates, one option for managing the lifelong burden of malabsorptive complications - such as osteoporosis, iron-deficiency anemia, and other catastrophic outcomes - is the surgical conversion of a Scopinaro BPD to an RYGB. This revision can help restore the absorptive capacity of the intestine [7].

In a 2022 study, Volpe et al. analyzed late postoperative complications in a cohort of 1,570 patients who had undergone a BPD with gastric preservation. Patients were followed for up to 20 years postoperatively. The results showed that 6.05% of patients who underwent the Scopinaro procedure suffered from long-term malnutrition. Of this malnourished group, 71.5% developed associated anemia, which required surgical revision, such as a common limb elongation or conversion to a gastric bypass [9].

In a literature review of 24 patients who had undergone BPD, only three had normal vitamin D and calcium levels. After revisional surgery (eight patients underwent revisional surgery), these patients experienced a decline in weight, along with a reduction in PTH levels and an increase in both vitamin D and calcium levels. It was also noted that weight loss was inversely associated with an increase in vitamin D and directly associated with a change in PTH [10].

## Conclusions

This case highlights the long-term nutritional complications associated with Scopinaro BPD, particularly severe hypocalcemia and vitamin D deficiency. Despite oral supplementation, the patient experienced recurrent hospital admissions and persistent symptoms due to chronic malabsorption. Surgical conversion to an RYGB resulted in significant clinical improvement, stabilization of calcium and Hgb levels, and resolution of symptoms. This underscores the importance of long-term follow-up for patients undergoing malabsorptive bariatric procedures. Early recognition and timely surgical intervention may help prevent irreversible complications and improve the quality of life for patients suffering from severe postoperative nutritional deficiencies.
